# Anxiety and depression among patients with age-related macular degeneration

**DOI:** 10.1192/j.eurpsy.2025.532

**Published:** 2025-08-26

**Authors:** S. V. Kuzmina, D. A. Yakovlev

**Affiliations:** 1 Psychiatry and medical psychology, Kazan State Medical University, Kazan, Russian Federation

## Abstract

**Introduction:**

AMD is currently the main cause of deterioration in the quality of life, disability and blindness in people over the age of 50 in economically developed countries,the presence of symptoms of anxiety and/or depression may be a risk factor for the development and exacerbation of ophthalmological diseases, a risk factor for an unfavorable prognosis of the disease and one of the factors contributing to a decrease in the patient’s compliance and motivation for ophthalmological treatment

**Objectives:**

The aim was to study the prevalence of symptoms of anxiety and depression in patients with age–related macular degeneration and determine its impact on quality of life.

**Methods:**

A continuous sampling method was used to examine 24 patients (9 men and 15 women, aged from 41 to 87 years, the average age in the group was 69.7 ± 10.8 years) with an established diagnosis of AMD, who were undergoing inpatient treatment at the Cheboksary branch of the FSAU NMIC MNTC Eye Microsurgery. Academician S.N. Fedorova” Ministry of Health of Russia, HADS, The Spielberger anxiety Questionnaire, SF-36,The results of the examination of the mental state were compared with the indicators of visual acuity

**Results:**

Clinically significant severity of depression symptoms was found in 4.2% of patients, mild manifestations of depression were found in 8.3%, 79.2% demonstrated moderate reactive anxiety; 12.5% of the subjects had a low level of reactive anxiety. 81.7 % of the respondents were subject to moderate and high personal anxiety. men were more prone to manifestations of both reactive (88.9% of men and 73.3% of women, respectively) and personal anxiety (100% of men and 86.7% of women, respectively), higher incidence of depression among women than among men. Patients with lower visual acuity tended to give a lower assessment of their physical health.

**Conclusions:**

The results demonstrated a high prevalence of personality and reactive anxiety among patients with age-related macular degeneration. At the same time, persons with higher acuity vision was more prone to anxiety, which may probably be due to incomplete adaptation to pathology in the early stages of AMD development. A tendency was found to have a worse assessment of their physical well-being among older patients with lower visual acuity.

This study has a limitation due to the small sample of patients at the time of analysis, which dictates the need for further study of this issue.

**Disclosure of Interest:**

None Declared

